# Influence of Spinal Movements Associated with Physical Evaluation on Muscle Mechanical Properties of the Lumbar Paraspinal in Subjects with Acute Low Back Pain

**DOI:** 10.3390/diagnostics12020302

**Published:** 2022-01-25

**Authors:** Sandra Alcaraz-Clariana, Lourdes García-Luque, Juan Luis Garrido-Castro, Cristina Carmona-Pérez, Daiana Priscila Rodrigues-de-Souza, César Fernández-de-las-Peñas, Francisco Alburquerque-Sendín

**Affiliations:** 1 Department of Nursing, Pharmacology and Physical Therapy, Faculty of Medicine and Nursing, University of Córdoba, 14004 Córdoba, Spain; m72alcls@uco.es (S.A.-C.); lgarcial05@hotmail.com (L.G.-L.); z62capec@uco.es (C.C.-P.); drodrigues@uco.es (D.P.R.-d.-S.); falburquerque@uco.es (F.A.-S.); 2 Department of Computer Science and Numerical Analysis, Rabanales Campus, University of Córdoba, 14071 Córdoba, Spain; cc0juanl@uco.es; 3 Maimonides Biomedical Research Institute of Cordoba (IMIBIC), 14004 Córdoba, Spain; 4 Department of Physical Therapy, Occupational Therapy, Rehabilitation and Physical Medicine, Universidad Rey Juan Carlos, Alcorcón, 28922 Madrid, Spain; 5 Cátedra Institucional en Docencia, Clínica e Investigación en Fisioterapia: Terapia Manual, Punción Seca y Ejercicio Terapéutico, Universidad Rey Juan Carlos, Alcorcón, 28922 Madrid, Spain

**Keywords:** myotonometry, thixotropy, viscoelasticity, spinal pain

## Abstract

This research aimed to identify changes in muscle mechanical properties (MMPs) when a standardized sequence of movements is performed and to determine the influence of acute low back pain (LBP) and age on the MMPs. Socio-demographic, clinical variables and MMPs were collected in 33 patients with LBP and 33 healthy controls. A 2 × 2 × 2 (group × age × time) analysis of variance (ANOVA) mixed model was used to determine the effect of the study factors on the different MMPs. There were no significant triple interactions. After the movements, tone and stiffness increased 0.37 Hz and 22.75 N/m, respectively, in subjects <35 years, independent of their clinical status. Relaxation showed differences by age in healthy subjects and creep in LBP subjects. Furthermore, elasticity was higher in <35 years (*p* < 0.001) without the influence of any other factor. In conclusion, sequenced movements can modify tone and stiffness as a function of age, while age-associated changes in viscoelastic characteristics depends on pain but not on movements. The MMPs should be assessed, not only at the beginning of the physical examination at rest, but also along the patient’s follow-up, depending on their pain and age, in a clinical setting.

## 1. Introduction

Back pain is one of the most common musculoskeletal pain disorders, affecting 80% of the population at some point in their lives [1]. The increasing prevalence of low back pain (LBP) is extensively detailed in the literature [2,3], which shows the necessity of a better understanding of the acute stage of the pathology to improve diagnosis and treatment, as well as to minimize the socio-economic burden [4]. Researchers categorize about 85% of LBP as non-specific, without structural changes, inflammation, or specific underlying disease [5,6]. Nevertheless, LBP has been associated with changes in neuromuscular activity [7,8], decreased spinal mobility, limited lumbar muscle flexibility, and altered spinal kinematics [9]. Furthermore, a modification of the mechanical changes associated to within and between muscle activity redistribution in subjects with LBP has been described [6,7]. These changes determine a reduction in lumbar spine function and alteration of the muscle mechanical properties (MMPs) [9,10,11,12], as well as inadequate motor control [13].

The MMPs play a central role in several physiological and pathophysiological processes, suggesting a relationship between pain and muscle stiffness in different spinal pain disorders, including LBP [14,15]. However, previous studies have not described predictive models of the anisotropic viscoelastic responses of relaxed muscles under physiological conditions. The changes in muscle tissues that determine spinal stiffness are not fully understood, requiring further research [15,16]. One of the characteristics of muscle tissue that could modify its mechanical properties is thixotropy [17]. Thixotropy refers to the property of a tissue to become fluid under certain conditions, such as voluntary movement or passive muscle stretch, and to return to its primary characteristic at rest [18]. Thixotropic substances are, therefore, history-dependent and have a “memory time” [19,20]. Some researchers described this thixotropic behaviour as a “short-range stiffness”, which tends to reduce or disappear after a few repetitions of movement and reappear after resting in healthy subjects [21]. The influence of such viscoelastic properties on the determination of MMPs in acute LBP and their modifications associated with the spinal movements performed on the physical evaluation, age or sex are relevant from a clinical point of view.

New technologies, such as elastography, help to study the passive mechanical behaviour of skeletal muscles [22]. However, their high cost makes them inaccessible to a clinical setting, challenging the determination of the MMPs. Since a decade ago, the device MyotonPRO© emerged as a non-invasive and reliable alternative to assess and monitor MMPs with clinical applications Several studies have shown sufficient accuracy and precision to determine MMPs in spinal muscles [23,24,25,26] and other tissues and regions [23,27,28,29], although efforts to better determine the concurrent validity need further research [30]. Previous research has associated MMPs alterations, specifically an increase in stiffness and tone or a decrease in elasticity, and axial spondyloarthritis [24]. Moreover, aging is associated with structural tissue remodelling, which contributes to increased stiffness and tone, and decreased elasticity at the cervical spine, and also the extremities [31,32]. Age and gender can also influence measures of muscle tone in the orofacial musculature [33]. Likewise, changes in cervical and lumbar MMPs concerning position or movement have been identified [31,33,34,35,36]. However, the behaviour of these patterns in the central stabilizer system, such as the paraspinal muscles [12], remains unknown when the subjects develop a standardized sequence of spinal movements, as is performed during physical examination, which could improve the diagnosis and treatment processes.

Therefore, this study aimed to identify changes in paraspinal MMPs when submitted to a standardized sequence of lumbar movements and to determine the influence of lumbar pain and age in those changes.

## 2. Methods

An observational, test-retest study including subjects with LBP and healthy controls was performed. Participants were recruited with a non-probabilistic sampling of two centres, Physiobalance (private physiotherapy centre) and the Biosanitary campus of the University of Córdoba, Spain, from November 2018 to January 2021. Assessments were conducted between April 2019 and March 2021. The Córdoba Research Ethics Committee approved this project (reference number 4016/2018). All participants signed written informed consent.

### 2.1. Participants

Sixty-six subjects of both sexes participated in this study. Thirty-three of them presented acute LBP with less than four weeks of evolution [37] and a pain score of ≥3 on the visual analogue scale (VAS) [38]. The control group consisted of 33 healthy subjects, matched by sex, age (±3 years), and body mass index (BMI) (±3 kg/m^2^), without spinal pain in the last six months or any neurological or musculoskeletal pain disorder.

The exclusion criteria for both groups were history of traumatic lesions at the spine, scoliosis, spinal surgery, congenital deformity, inflammatory disease, pregnancy, and receiving spinal physiotherapy treatment in the last six months.

### 2.2. Sample Size

A moderate difference size (Cohen d = 0.7) [39], considered clinically relevant in musculoskeletal pathology and physiotherapy field [40], was estimated for between-group comparisons of MMPs. With an alpha level of 0.05 and power of 0.8, 33 subjects per group were needed (GPower 3.1 software, Düsseldorf, Germany).

### 2.3. Assessments and Procedures

After signing the informed consent form, researchers collected socio-demographic (age, sex, weight, height, and BMI) and clinical (severity of back pain and disability using a VAS scale and the Oswestry Disability Questionnaire (ODI) data, respectively [41]). Subsequently, the MMPs, using a MyotonPRO^®^ (Myoton AS, Tallinn, Estonia) device, were assessed before and after a standardized sequence of movements. The MyotonPRO© provides measurements of five MMPs: tone or state of tension, determined by the frequency (Hz); biomechanical properties such as dynamic stiffness (N/m) and decrement, that characterizes elasticity; and viscoelastic properties such as relaxation or mechanical stress relaxation time (ms) and creep (Deborah number), which corresponds to the gradual elongation in the tissue when subjected to constant tension [42]. The device induces a natural damped oscillation of the tissue following the application of a controlled load through a cannula. To the initial 0.18 N of compression of the subcutaneous tissue, the MyotonPRO^®^ added a pulse of 15 ms and 0.40 N of mechanical force. The accelerometer at the sensor’s tip provides the data that characterizes the tissue [43].

**Figure 1:**
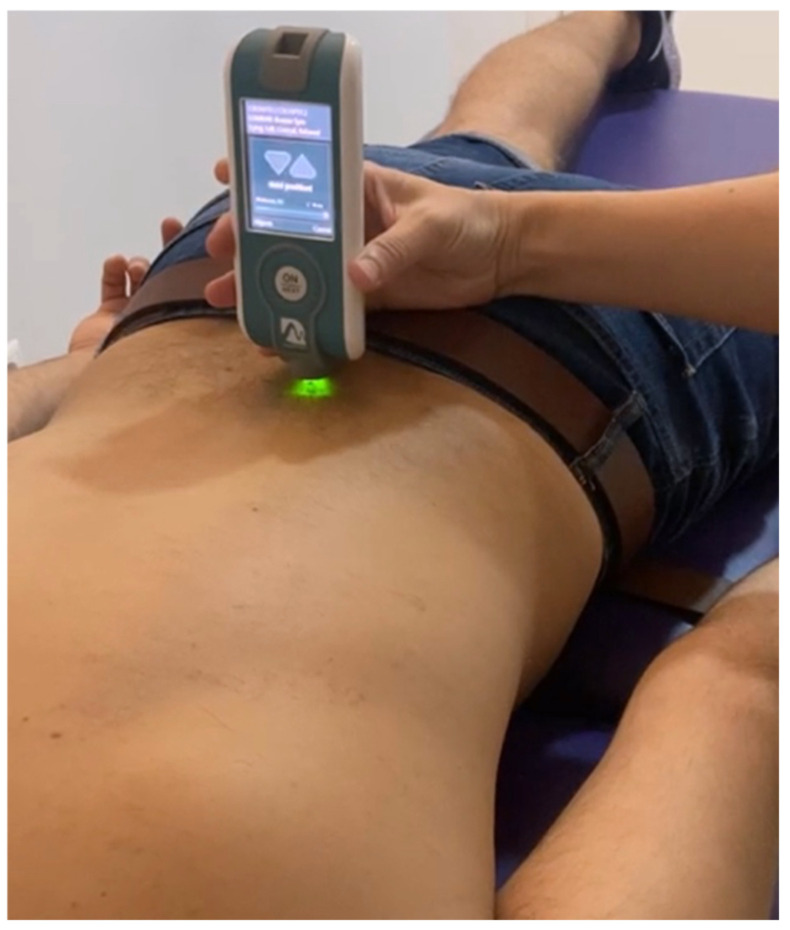
Procedure for measuring MMPs at lumbar level with MyotonPRO©.

A clinician with more than fifteen years of clinical experience, identified the spinous process of L5 by palpatory testing and performed the tonometric test on the erector spinae, located 2.5 cm to the right and left of the spinous process. The assessment was performed with the patient in a prone position with both arms alongside the body. Muscle measurements were taken first on the left side, then on the right side, and the process was repeated. The measurement was taken during a five-second apnoea after the normal expiratory phase [24,44] (Figure 1).

The standardized sequence of movements involved the maximum range of motion in frontal flexion, extension, lateral flexion, and rotation of the spine in a standing position, all routinely used in the conventional evaluation of the amount of movement of patients in a clinical setting [45]. Each movement lasted four seconds, with two seconds to achieve the maximum range of motion and two seconds to return to the neutral position, controlled with a metronome (view Supplementary Material, Figures S1–S6). A total of three repetitions per movement were performed, supervised by a second clinician with more than ten years of clinical experience. The whole procedure did not take more than ten minutes.

### 2.4. Statistical Analysis

Descriptive results for qualitative variables were expressed as frequencies and percentages. Quantitative variables were expressed as means, standard deviations, and 95% confidence interval (CI). The Kolmogorov–Smirnov test was used to assess the normality of the data distribution, with *p* > 0.05 in all cases.

In a preliminary analysis of the data, no side-to-side differences were identified when applying Student’s *t*-test between measurements on each group (*p* > 0.05). Consequently, pooled (mean) data from both sides were used in the main analysis.

A 2 × 2 × 2 mixed model (group × age × time) of analysis of variance (ANOVA) was used to determine the effect of the study factors on the different MMPs. The first factor was clinical status (LBP patients vs. healthy subjects). The second factor was the age, with two levels (subjects <35 vs. >35 years) as previous studies with similar objectives has defined [1,46,47,48]. Finally, the third factor analysed was time of assessment, with before and after movement measure as the levels of this repeated measures factor. The first hypothesis of interest was the triple interaction. In the absence of triple interaction, double interactions (group-by-time, group-by-age, time-by-age) were those of interest. If no interactions were observed, the main effect of each factor was finally studied. Pairwise comparisons were conducted by post hoc Bonferroni tests when necessary.

In all cases, the confidence level was established at 95%, and the statistical significance level for the tests was *p* < 0.05. The analyses were carried out by IBM SPSS^®^ Statistics version 25 (SPSS Inc., Chicago, IL, USA).

## 3. Results

A total of 66 subjects were analysed, 33 with acute LBP (<35 years *n* = 19, >35 years *n* = 14) and 33 healthy controls (<35 years *n* = 18, >35 years *n* = 15), with a mean age of 33.3 ± 11.8 years and a BMI of 23.9 ± 2.6 kg/m^2^ (Table 1).

There was no triple interaction when analysing the pooled effect of movement, clinical status, and age on the behaviour of the MMPs (frequency: F = 0.169, *p* = 0.682; stiffness: F = 0.623, *p* = 0.433; decrement: F = 0.947, *p* = 0.334; relaxation: F = 0.003, *p* = 0.982; creep: F = 0.632, *p* = 0.430).

With borderline statistical significance found in the double interactions, frequency (F = 3.342, *p* = 0.045) and stiffness (F = 3.145, *p* = 0.048) increased 0.37 Hz (95% CI 0.06–0.70) and 22.75 N/m (95% CI 5.83–39.67), respectively, in the younger subjects after the movements, independent of their clinical status. Only the baseline measure showed significant differences (*p* < 0.05) between both age groups in both MMPs. No other time-by-age interaction was identified neither for decrement (F = 0.060, *p* = 0.808), relaxation (F = 1.599, *p* = 0.211), and creep (F = 0.007, *p* = 0.934) outcomes.

Likewise, the relaxation and creep showed interactions between age and clinical status of the subjects (F = 3.202, *p* = 0.047; F = 3.345, *p* = 0.045, respectively), with relaxation being 2.98 ms (95% CI 0.36–6.01) higher in young healthy subjects. In contrast, creep was 0.10 (95% CI 0.06–0.15) greater in subjects with LBP over 35 years. Differences between patients and controls in those over 35 were also found (mean difference 0.12, 95% CI 0.01–0.27, *p* = 0.04). Frequency (F = 1.391, *p* = 0.243), stiffness (F = 0.675, *p* = 0.414), and decrement (F = 0.013, *p* = 0.908) did not show group-by-age interaction.

The group-by-time interaction did not reveal any statistical significance (frequency: F = 0.244, *p* = 0.623; stiffness: F = 0.028, *p* = 0.868; decrement: F = 0.212, *p* = 0.647; relaxation: F = 0.135, *p* = 0.714; creep: F = 0.111, *p* = 0.740).

Finally, the evaluation of the main factors showed that the decrement was different depending on age. Thus, older subjects presented higher decrement than the younger ones (mean difference 0.337, 95% CI 0.21–0.46, F = 29.176, *p* < 0.001) (Table 2).

## 4. Discussion

The results showed that paraspinal lumbar MMPs have different behaviour under specific conditions of lumbar movements, clinical status or age. Thus, the sequenced lumbar movements protocol influenced tone and stiffness depending on age, with lower values for the MMPs in younger subjects, which were more susceptible to be influenced by movement. However, the presence of LBP did not influence MMPs. On the other hand, although with almost statistical significance, the viscoelastic state of paraspinal muscles, expressed as relaxation and creep, depends on the combination of pain and age. While healthy subjects showed a reduction in relaxation with age, these differences did not occur in individuals with LBP. The presence of pain determined the differences in creep concerning age. In this case, the performance of sequenced lumbar movements did not influence the MMPs. Furthermore, the elasticity, as the inverse of the decrement, was higher in younger subjects, without the influence of any other factor.

Curiously, when the presence or absence of LBP was analysed separately, no significant differences were detected, despite the higher values of tone, stiffness, and lower values of decrement, relaxation, and creep found in LBP subjects. This was an unexpected result, since acute LBP is commonly associated to muscle spasm throughout the paraspinal muscles [13,49,50]. However, it could be explained by the mild disability that showed the current sample. Finally, it is relevant to note that there was no interdependence among the execution of the movements, the age, and the clinical condition of the subjects, showing that the three factors were independent. In summary, the influence of movements, pain, and age is different depending on each specific MMP, which means that MMPs should be assessed, not only at the beginning of the physical examination, but also along the follow-up of the patient, with emphasis on elder subjects and those with pain.

This study determined the interaction between the effect of sequenced movements and the advancing of age on tone and stiffness at the lumbar level, where the first measure reports a difference between age groups of 1.29 Hz that decreases to 0.88 Hz after movement. This difference indicates that younger subjects experienced a change in their muscle tone that did not occur in older subjects. Regarding stiffness, from a difference between age groups of more than 50 N/m, younger subjects increased their stiffness more than 8% after the movements, while older subjects showed a rise of only 2%. Apparently, our results would contradict the behaviour of thixotropic properties of specific tissues, which establishes the reduction of viscosity and, therefore, of muscle stiffness, during and, for some time, after movement [19,21,51]. However, the procedures to perform the movements associated to physical evaluation may explain the results. In fact, Altman et al. [18] reported that the frequencies of movements that allow observing both thixotropic and rheopectic reactions, this one being opposite phenomenon to thixotropy, in muscle fibres are in the range of 1 to 20 Hz [20]. Specifically, in those movements below 1 Hz, a rheopectic behaviour is observed in activated fibres in rabbits [18]. Likewise, although some authors report thixotropic behaviours, even in short-range movements or with just two repetitions [21,51], the recovery of rigidity could occur after only 15 s of rest [21]. This could explain our observations, because the lumbar movements associated with physical evaluation have a low frequency and are followed by rest periods.

On the other hand, the fact that only subjects under 35 years presented a significant change in muscle tone and stiffness after movement supports the relationship between age and MMPs changes [14,31,32,44]. Supporting this approach, Ayapong-Badu et al. [32] identified an 30% decrease in elasticity between age groups for biceps brachialis and between 20% and 30% for rectus femoris, as was detected in our sample in the lumbar muscles, and changes in tone of 10%. With similar aims, Kocur et al. [31] showed that aging provokes an increase in the tone and stiffness of 11–17% for sternocleidomastoid and trapezius muscles in healthy subjects. These changes in MMPs could be due to the muscle composition and architecture alterations that occur with advancing age [31,33,52] and begin in the young adulthood [53,54]. For example, some authors have suggested that increased intramuscular adipose tissue in older adults is the cause of increased muscle stiffness [52,55], as well as the qualitative change in muscle fibres, with an increase in the proportion of type I muscle fibres, characterized by greater stiffness than type II ones [31]. Curiously, the tendon tissue shows a decrease in tone and stiffness in elderly subjects [56,57], which means that different tissues show specific physiological adaptations to age. In summary, for evaluation purposes, the significant changes in tone and post-movement stiffness in subjects under 35 years suggest that the baseline measure represents the best approach to characterize the MMPs in this population.

Our study also reported the relationship between LBP and age with relaxation, and creep, as viscoelastic characteristics, of paraspinal muscles. Relaxation was different in healthy subjects depending on age. In fact, advanced age established differences in creep between healthy subjects and patients. However, the presence of LBP inhibited this behaviour in the creep. Although there is no previous research on viscoelastic properties in acute LPB, previous studies have determined changes in MMPs in subjects with chronic mechanical and inflammatory LBP and neck pain [24,36,43,58]. In these studies, the differences for MMPs between subjects with spinal pain and healthy subjects were explained by the response of muscle spasm to pain, which decreases circulation and increases stiffness [50], disuse as a cause of muscle atrophy, that also increases stiffness [33], or disease of long duration, that alters elasticity and stiffness [43]. It is possible that the acute LBP and, consequently, the short period of evolution of our sample, prevented the changes in the MMPs. Future studies are required to confirm these findings as well as determine these effects on a longer follow-up.

### Strengths and Limitations

To date, no previous study has attempted to establish the adequate moment to evaluate MMPs in clinical practice based on the physical evaluation, clinical status and age. Consequently, these results will allow us to determine the effect of the therapy on the MMPs with greater precision.

However, several limitations must be recognized. First, the raters were not blinded, although the assessment of the MMPs has shown high reliability and low rater dependence, which limits a negative influence on the results [25,59]. Second, the evaluation of the MMPs was only determined in paraspinal muscles at the L5 level, making it impossible to know if other muscles and tissues exhibit similar behaviour. Third, although 35 years has been used to distinguish younger than older adults [32,46,47], other age classifications could provide different results and interpretations. Additionally, the study of other factors, such as the level of physical activity, work or leisure-time activity [60,61,62], among others, could have afforded other relationship patterns to the results. Furthermore, adding other techniques to analyse muscle characteristics, such as surface electromyography, could provide information on normal or unusual activity, such as muscle spasm during the evaluation. Finally, our sample showed mild disability according to ODI, which could limit the external validity of the results for higher levels of disability.

## 5. Conclusions

A sequenced spinal movement protocol can modify lumbar paraspinal tone and stiffness according to the patient’s age but not according to the presence of LBP. The changes in viscoelastic characteristics of lumbar muscles depend on age and the presence or absence of pain, but not on the performance of the sequenced movements. Older subjects showed less elasticity than younger ones at the L5 spinal level, independent of their condition.

The MMPs should be assessed in a clinical setting, not only at the beginning of the physical evaluation during rest, but also during the patient’s follow-up, with special attention to elder subjects and those with pain.

## Figures and Tables

**Table 1:** Demographic characteristics of the sample.

	LBP Group (N = 33)	Healthy Group (N = 33)	*p*-Value
Age (years)	33.05 ± 11.8	33.6 ± 12.0	NS
Sex (female/male)	14/19	14/19	NS
BMI (kg/m^2^)	24.2 ± 2.45	23.6 ± 2.8	NS
Pain intensity (VAS)	4.7 ± 1.7	-	-
ODI	7.8 ± 5.4	-	-

Results are expressed as: mean ± standard deviation, frequencies. Abbreviations: BMI: Body mass index, VAS: Visual analogic scale, ODI: Oswestry disability questionnaire, NS: not significant differences.

**Table 2:** Results of MMPs according to the clinical status (LBP group, N = 33; healthy group, N = 33), age, (under 35 years, *n* = 37; over 35 years, *n* = 29), and time of assessment.

	Group	Age	Baseline Evaluation	After Movement Evaluation	Between Evaluation Differences
Frequency (Hz)	LBP	<35 years	14.26 ± 1.50	14.64 ± 2.07	0.38 (0.83, −0.06)
	Healthy		13.94 ± 1.40	14.30 ± 1.87	0.36 (0.82, −0.09)
	Between clinical status differences		0.31 (−0.98, 1.61)	0.33 (−1.09, 1.77)	
	LBP	>35 years	14.91 ± 2.51	14.98 ± 2.50	0.07 (0.59, −0.44)
	Healthy		15 87 ± 2,46	15.73 ± 2.33	−0.14 (0.35, −0.64)
	Between clinical status differences		−0.96 (−2.43, 0.50)	0.74 (−2.36, 0.87)	
Stiffness (N/m)	LBP	<35 years	250.26 ± 59.63	269.03 ± 89.23	18.77 (42.37, −4.82)
	Healthy		235.23 ± 55.73	261.95 ± 110.83	26.72 (50.97, 2.47)
	Between clinical status differences		15.02 (−30.99, 61.05)	7.08 (−53.09, 67.26)	
	LBP	>35 years	283.36 ± 80.95	295.48 ± 76.09	12.12 (39.61, −15.37)
	Healthy		309.95 ± 85.27	309.85 ± 81.04	−0.10 (26.46, −26.66)
	Between clinical status differences		−26.58 (−78.58, 25.41)	14.36 (−82.35, 53.62)	
Decrement	LBP	<35 years	1.05 ± 0.23	1.08 ± 0.31	0.02 (0.10, −0.05)
	Healthy		1.08 ± 0.22	1.04 ± 0.17	−0.03 (0.04, −0.11)
	Between clinical status differences		−0.02 (−0.19, 0.15)	0.04 (−0.13, 0.21)	
	LBP	>35 years	1.41 ± 0.24	1.41 ± 0.21	−0.00 (0.09, −0.09)
	Healthy		1.38 ± 0.34	1.40 ± 0.33	0.01 (0.10, −0.07)
	Between clinical status differences		0.03 (−0.16, 0.22)	0.01 (−0.18, 0.21)	
Relaxation (ms)	LBP	<35 years	19.35 ± 5.89	19.05 ± 5.96	−0.29 (0.38, −0.98)
	Healthy		20.79 ± 4.05	20.35 ± 4.61	−0.44 (0.28, −1.17)
	Between clinical status differences		−1.44 (−4.56, 1.66)	−1.30 (−4.54, 1.94)	
	LBP	>35 years	19.26 ± 4.20	19.43 ± 4.54	−0.16 (0.96, −0.63)
	Healthy		17.58 ± 3.86	17.61 ± 3.70	0.03 (0.80, −0.73)
	Between clinical status differences		1.68 (−1.77, 5.15)	1.81 (−1.79, 5.43)	
Creep (Deborah Number)	LBP	<35 years	1.15 ± 0.14	1.13 ± 0.19	−0.02 (0.05, −0.09)
	Healthy		1.20 ± 0.22	1.16 ± 0.28	−0.04 (0.035, −0.11)
	Between clinical status differences		−0.04 (−0.19, 0.09)	0.03 (−0.19, 0.12)	
	LBP	>35 years	1.27 ± 0.28	1.22 ± 0.28	−0.05 (0.02, −0.14)
	Healthy		1.14 ± 0.21	1.12 ± 0.18	−0.01 (0.07, −0.09)
	Between clinical status differences		0.13 (−0.25, 0.30)	0.09 (−0.08, 0.27)	

Results are expressed as mean ± standard deviation, mean difference (95% confidence interval).

## Data Availability

The data presented in this study are available upon reasonable request from the corresponding author.

## Supplementary Materials

The following are available online at https://www.mdpi.com/article/10.3390/diagnostics12020302/s1, Figure S1: Flexion, Figure S2: Extension, Figure S3: Right lateral flexion, Figure S4: Left lateral flexion, Figure S5: Right rotation, Figure S6. Left rotation.
