# Supplementary material for: Influence of Spinal Movements Associated with Physical Evaluation on Muscle Mechanical Properties of the Lumbar Paraspinal in Subjects with Acute Low Back Pain
Source: Diagnostics (Basel). 2022 Jan 25;12(2):302. doi: 10.3390/diagnostics12020302 (PMC8870934; doi:10.3390/diagnostics12020302)
Supplement: Supplementary file 1 [file diagnostics-12-00302-s001.zip › diagnostics-1543068-supplementary.pdf]

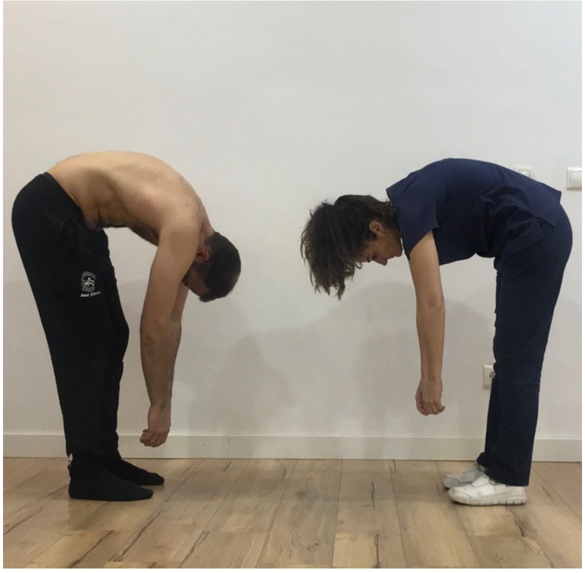

Figure S1. Flexion

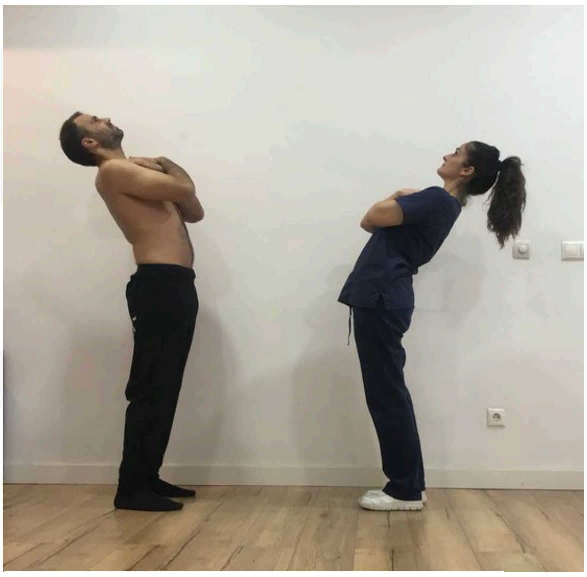

Figure S2. Extension

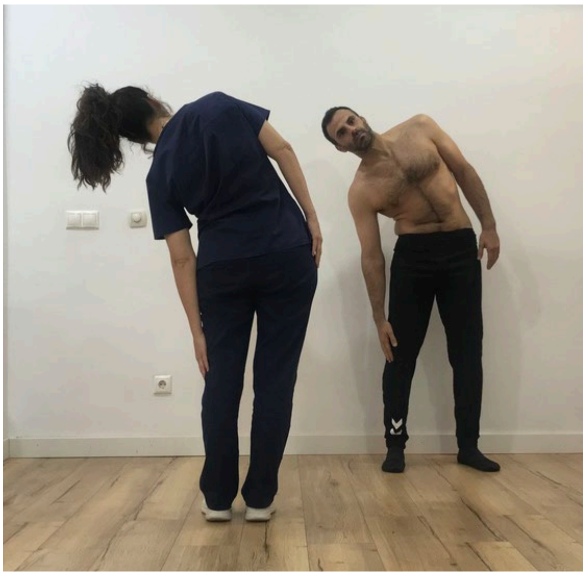

Figure S3. Right lateral flexion

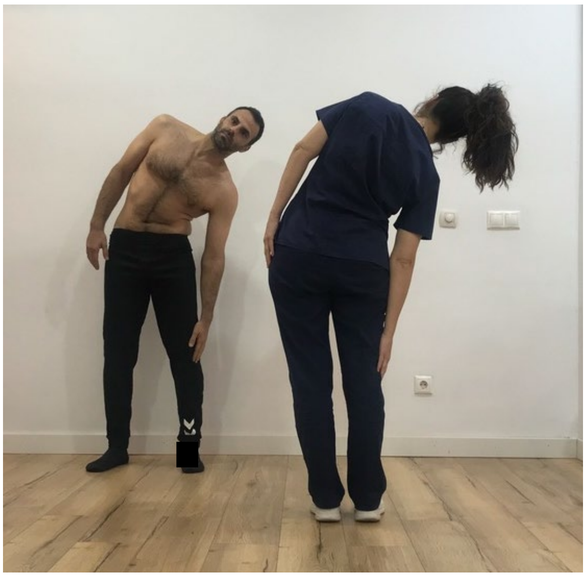

Figure S4. Left lateral flexion

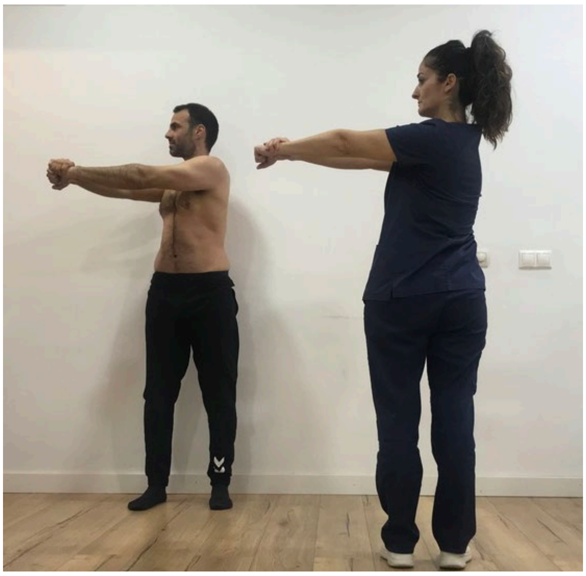

Figure S5. Right rotation

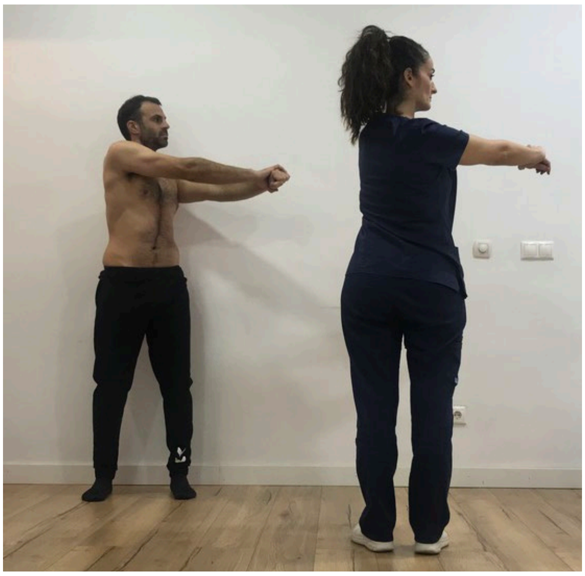

Figure S6. Left rotation
